# Investigating cyclic nucleotide and cyclic dinucleotide binding to HCN channels by surface plasmon resonance

**DOI:** 10.1371/journal.pone.0185359

**Published:** 2017-09-26

**Authors:** Sebastien Hayoz, Purushottam B. Tiwari, Grzegorz Piszczek, Aykut Üren, Tinatin I. Brelidze

**Affiliations:** 1 Department of Pharmacology and Physiology, Georgetown University Medical Center, Washington, DC, United States of America; 2 Department of Oncology, Georgetown University Medical Center, Washington, DC, United States of America; 3 Biophysics Core, National Heart, Lung and Blood Institute, National Institutes of Health, Bethesda, MD, United States of America; Universitat Regensburg, GERMANY

## Abstract

Hyperpolarization-activated cyclic nucleotide-modulated (HCN) channels control cardiac and neuronal rhythmicity. HCN channels contain cyclic nucleotide-binding domain (CNBD) in their C-terminal region linked to the pore-forming transmembrane segment with a C-linker. The C-linker couples the conformational changes caused by the direct binding of cyclic nucleotides to the HCN pore opening. Recently, cyclic dinucleotides were shown to antagonize the effect of cyclic nucleotides in HCN4 but not in HCN2 channels. Based on the structural analysis and mutational studies it has been proposed that cyclic dinucleotides affect HCN4 channels by binding to the C-linker pocket (CLP). Here, we first show that surface plasmon resonance (SPR) can be used to accurately measure cyclic nucleotide binding affinity to the C-linker/CNBD of HCN2 and HCN4 channels. We then used SPR to investigate cyclic dinucleotide binding in HCN channels. To our surprise, we detected no binding of cyclic dinucleotides to the isolated monomeric C-linker/CNBDs of HCN4 channels with SPR. The binding of cyclic dinucleotides was further examined with isothermal calorimetry (ITC), which indicated no binding of cyclic dinucleotides to both monomeric and tetrameric C-linker/CNBDs of HCN4 channels. Taken together, our results suggest that interaction of the C-linker/CNBD with other parts of the channel is necessary for cyclic-dinucleotide binding in HCN4 channels.

## Introduction

The mammalian hyperpolarization-activated cyclic nucleotide-modulated (HCN) family of channels contains four subfamilies, HCN1-HCN4 [1–4]. HCN channels are widely expressed in the mammalian brain [5–7] and heart [8–10], where they generate I_h_ (hyperpolarization) and I_f_ (funny) currents, respectively. Among the four subfamilies HCN2 channels are the most prevalent in the brain and HCN4 account for more than 80% of the total HCN mRNA in the heart [1]. Due to their unique activation mechanism by membrane hyperpolarization HCN channels are the major contributors to the rhythmic firing of neurons and cardiac myocytes [1,11,12]. HCN channels also contribute to setting the neuronal resting potential and dendritic integration [1].

**Fig 1:**
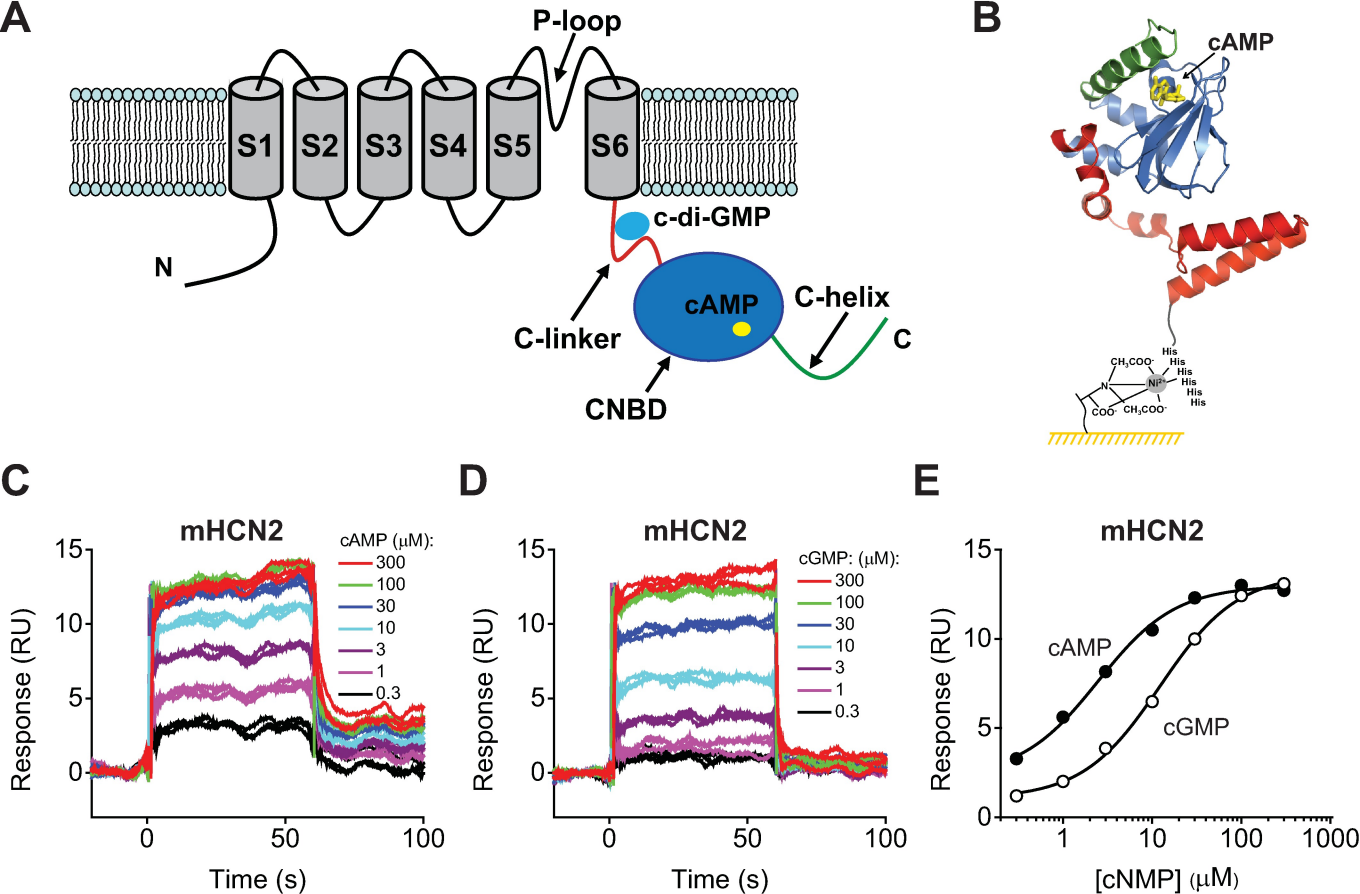
Detecting cNMP binding to the HCN2 C-linker/CNBDs with SPR. (A) Cartoon of one of the four subunits of HCN channels. The transmembrane segments S1-S6 are grey, the C-linker is red, the CNBD domain is blue and the C-helix is green. cAMP bound to the CNBD and c-diGMP bound at the proposed site formed by the C-linker are depicted by yellow and cyan ellipses. (B) Cartoon of the mHCN2 C-linker/CNBD immobilized on the NTA chip surface using Ni^2+^-NTA and the N-terminal 6-His tag coupling. In the ribbon representation of the C-linker/CNBD [15], the C-linker is red, the β-roll and helices A, P and B are blue, and the C-helix is green. cAMP bound inside the β-roll cavity is yellow. (C) and (D) Representative SPR sensorgrams recorded for the immobilized HCN2 C-linker/CNBDs with the indicated concentrations of cAMP (C) and cGMP (D). C-linker/CNBDs immobilized on the same surface were used in (C) and (D). (E) Plots of the SPR response at 30 s after the start of the injection versus total cAMP (filled circles) and cGMP (open circles) concentration for sensorgrams shown in (C) and (D). The lines represent fits of the data with Hill equation. The binding affinities were 2.5 ± 0.4 μM for cAMP and 12.3 ± 1.1 μM for cGMP.

HCN channels are tetramers with each subunit containing six transmembrane segments (S1-S6) and an intervening P-loop (Fig 1A) [3,4]. The S1-S4 segments comprise a voltage sensor, while the S5-S6 segments together with the P-loop form a centrally located pore of the channel. The characteristic feature of HCN channels is the presence of the cyclic nucleotide-binding domain (CNBD) in their C-terminal region. The CNBD, which contains the ‘canonical’ cyclic nucleotide binding site, consists of four α-helices (A, P, B, C) and a β-roll formed by eight β-strands flanked by the A- and B-helices (Fig 1B) [13–15]. The CNBD is connected to the pore forming transmembrane segment via the C-linker. The opening of HCN channels is facilitated by direct binding of cyclic nucleotides to the CNBD (reviewed in [1]). With a combination of structural, functional and fluorescence-based studies, and molecular dynamics simulations a chain of events leading to the channel opening is beginning to emerge [16–20]. Binding of cyclic nucleotides to the CNBD causes the C-helix to move closer to the β-roll [16–20]. It is though that this initial structural rearrangement promotes tetramerization of the C-linker/CNBDs [15,21] and causes a centrifugal movement of the C-linker that widens the pore, facilitating the channel opening [22].

Recently, Lolicato et al. reported that cyclic dinucleotides decrease the effect of cyclic nucleotides on the opening of HCN4 channels but not HCN2 channels [23]. Cyclic dinucleotides, ubiquitous signaling molecules in bacteria [24], are newly discovered second messengers in eukaryotes [25,26]. Although, the physiological importance of the cyclic dinucleotide regulation of HCN channels is not clear at this point, understanding the molecular mechanisms of this regulation is important for building a coherent picture of HCN channel function and might provide clues for the design of novel isoform-specific HCN channel regulators. The initial study by Lolicato et al. provided several insights on the molecular mechanisms of cyclic dinucleotide regulation, however, it also raised important questions. The initial hint on the cyclic dinucleotide regulation was obtained from the crystal structure of the isolated C-linker/CNBD of HCN4 channels crystallized in the presence of cGMP [23]. The crystal structure revealed cGMP bound to two sites, one ‘canonical’ site formed by the CNBD and the second site formed by the C-linker pocket (CLP) at the interface between the C-linker and CNBD. The CLP site was big enough to accommodate two cGMP molecules, prompting the authors to look into HCN4 channel regulation by cyclic dinucleotides. Based on the virtual docking of c-di-GMP inside the structure of the isolated C-linker/CNBD Lolicato et al., proposed that cyclic dinucleotides regulate HCN4 channels by binding to the CLP site. The authors did not provide a direct evidence of cyclic dinucleotide binding. However, the hypothesis of the cyclic dinucleotide binding to the CLP site was strengthened by mutational analysis of residues predicted to line the putative binding pocket, which revealed that R680E mutation completely abolished cyclic-dinucleotide modulation of HCN4 channels [23]. The C-linkers of the HCN2 and HCN4 channels are highly conserved in their amino acid sequence and have very similar structure when part of the isolated C-linker/CNBD protein (S1 Fig) [13–15,23]. Moreover, the R680 residue is also conserved in HCN2 channels. Therefore, the mechanism of the HCN4 isoform-specific modulation by cyclic dinucleotides is not clear.

Here we used surface plasmon resonance (SPR) method to investigate cyclic nucleotide and cyclic dinucleotide binding to the isolated C-linker/CNBDs of HCN2 and HCN4 channels. Using the SPR method we found that while cyclic nucleotides (cAMP and cGMP) bind to the immobilized C-linker/CNBDs of HCN4 and HCN2, cyclic dinucleotides do not bind to the immobilized C-linker/CNBDs of HCN4 and HCN2 channels. This result was further supported with isothermal calorimetry method that also showed no binding of cyclic dinucleotides to the isolated monomeric and tetrameric C-linker/CNBD of HCN4 channels. Taken together, our results suggest that the cyclic dinucleotide binding in HCN channels requires coupling of the C-linker to the rest of the channel.

## Materials and methods

### Protein expression and purification

For surface plasmon resonance (SPR) and isothermal calorimetry (ITC) based experiments at low concentrations of the protein, C-linker/CNBDs of the wild-type and L586W mutant mouse HCN2 (mHCN2, residues 443–645) and human HCN4 (hHCN4, residues 521–723) channels were subcloned into pETM11 bacterial expression vector containing an N-terminal 6-His affinity tag followed by a tobacco etch virus protease (TEV) cleavage site. The proteins were expressed in BL21 (DE3) cells, and purified with Ni^2+^-NTA and size exclusion chromatography, as previously described [27,28]. Briefly, bacterial cultures were grown at 37 ^o^C to the OD of 0.6–0.8 and induced with 1 mM isopropyl β-D-1-thiogalactopyranoside (IPTG) at 18 ^0^C overnight. The cells were harvested by centrifugation and re-suspended in buffer A (150 mM KCl, 10% Glycerol, 1 mM tris(2-carboxyethyl)phosphine (TCEP), 30 mM HEPES; pH 7.5), supplemented with 1 mM 4-(2-aminoethyl) benzenesulphonyl fluoride hydrochloride (AEBSF) and 2.5 mg/ ml DnaseI. Cells were lysed with an EmulsiFlex C-5 homogenizer (Avestin). Insoluble protein was separated by centrifugation at 30,000 rpm for 1 hr at 4°C in a Beckman 45Ti rotor and the supernatant was loaded onto His-Trap HP column (GE Healthcare). The column was washed with buffer A and the proteins were eluted with buffer A + 500 mM imidazole. The proteins were further purified with size exclusion chromatography on a Superdex 200 Increase column (GE Healthcare) equilibrated with buffer A.

Testing cyclic dinucleotide binding to a tetrameric C-linker/CNBD of hHCN4 channels required high concentrations of the protein, which were not achievable for the C-linker/CNBD when expressed as a 6-His tagged protein in pETM11 vector. To increase protein yield the C-linker/CNBD of hHCN4 (residues 521–723) channels was subcloned into pHMALc2T bacterial expression vector containing an N-terminal maltose binding protein (MBP) tag followed by a thrombin cleavage site. The protein was expressed in BL21 (DE3) cells. The cells were harvested by centrifugation, re-suspended in the lysis buffer, lysed with an EmulsiFlex C-5 homogenizer and insoluble protein was separated by centrifugation in the same manner as for the C-linker/CNBDs in pETM11 vector. The supernatant was loaded onto MBPTrap HP column (GE Healthcare). The column was washed with buffer A and the protein was eluted with buffer A + 50 mM maltose. The MBP tag was removed by thrombin cleavage for 4 hrs at RT. The salt concentration was lowered by the addition of a no-salt buffer B (30 mM HEPES, 1 mM TCEP, pH 7.0) and the cleavage reaction was loaded onto HiTrap SP FF column (GE Healthcare). The protein was eluted with a gradient of 0.1–1 m KCl. Monodispersity of the protein was confirmed with size-exclusion chromatography on a Superdex 200 Increase column (GE Healthcare) equilibrated with buffer A.

The protein concentrations were determined with Coomassie (Bradford) Protein Assay (ThermoFisher Scientific). For SPR experiments and ITC experiments at low concentrations of the C-linker/CNBDs the purified proteins were stored at -80 ^o^C in small aliquots before use. For ITC experiments at high concentrations of the C-linker/CNBDs purified proteins were used immediately after the final purification step. The molecular weight of the purified proteins was verified with mass spectrometry (electrospray) at Georgetown Proteomics and Metabolomics Core Facility.

### Surface plasmon resonance measurements

SPR method is based on the phenomenon of surface plasmons, electron charge density waves that propagate along the interface between two media [29,30]. For the Biacore system used in our study the interface is between the glass of the sensor chip and the sample solution, with a thin conducting layer of gold separating the two media. Under conditions of total internal reflection, an incident light that strikes the chip surface is reflected without losing net energy. However, at a certain resonance angle, called the SPR angle, a characteristic absorption of energy happens that is manifested as a sharp drop in the intensity of the reflected light. The resonance angle depends on the refractive index in the vicinity of the sensor surface. Binding of an analyte to a protein immobilized on the chip surface causes changes in the refractive index and is detected as a shift in the resonance angle. This response is reported in the resonance units (RU). 1 RU corresponds to 0.0001 degree of change in the reflected light angle.

SPR experiments were performed at 25 ^o^C on a Biacore 4000 Instrument (GE Healthcare). C-linker/CNBDs of wild-type and L586W mutant mHCN2, and hHCN4 channels were immobilized on a NTA chip (GE Healthcare), as previously described [31,32]. Immobilizations of the proteins were performed in HBS-P buffer (150 mM NaCl, 10 mM HEPES, 0.05% (v/v) surfactant P20, pH 7.4). First the NTA sensor surface was activated with a 1 min injection of 0.5 M NiCl_2_. The coupling of the Ni^2+^-NTA chip surface groups with the 6-His-tagged proteins was then achieved by 2.5 min injections of the proteins at 200 nM concentrations to the chip surface. After the initial capturing, the proteins were covalently cross-linked via 20 s injections of NHS-EDC carboxyl-reactive cross-linkers to prevent protein loss from the chip surface with successive analyte and buffer injections. This was followed by 20 s injection of 1 M ethanolamine to block the remaining reactive sites. The proteins were captured at ~1000–3000 resonance units (RU; 1 RU = 1 pg of protein per mm^2^). In all SPR experiments a reference spot was activated and blocked using similar coupling chemistry as for the active spots but with no proteins immobilized. This spot was used as a reference surface to account for a non-specific binding to the chip surface. The binding to the reference surface was subtracted from the binding to the surfaces with immobilized proteins. In addition, the binding corresponding to blank injections (buffer only) was subtracted from the reference subtracted SPR data.

The binding experiments were performed in the running buffer (150 mM KCl, 10% Glycerol, 1 mM TCEP, 30 mM HEPES, pH 7.5). Analytes over the range of concentrations were injected in triplicates over the chip surface for 60 s at a flow rate of 30 μl /min, followed by buffer only injections. The regeneration of the surface between the analyte injections was not necessary, as the baseline returned to the pre-injection levels. In some experiments we observed a drift in the baseline. This was because the reference subtraction described above was not perfect and resulted in the less than ideal correction. However, there was minimal residual binding between analyte injections, as indicated by essentially the same binding response for triplicate repeats of analyte injections at the same concentrations.

For the SPR experiments cAMP and cGMP were purchased from Sigma. c-di-GMP, c-di-AMP and 2’3’-cGAMP were purchased from BioLog Life Science Institute. Each experiment was repeated on at least three different NTA chips.

To determine the binding affinity (K_d_), the steady state SPR responses (R_eq_) at 30 s after the start of the injection were plotted against the analyte concentration and fitted with a Hill equation with Hill coefficient of 1, as described previously [33]. Analyte concentrations higher than 300 μM exhibited strong non-specific binding to the reference surface. Therefore, concentrations higher than 300 μM were excluded from the analysis. The data analysis and fitting of the plots was performed in Origin (Microcal Software, Inc). Error bars indicate the SEM. Omission of error bars indicates that the SEM was less than the size of the symbol in the figures. n represents the number of different NTA chips used for the same analyte injections. For each NTA chip an analyte was injected three times. Therefore, each experiment was repeated at least 3xn times. Statistical analysis was performed by using one-way ANOVA. P < 0.05 was considered statistically significant.

### Isothermal titration calorimetry

For ITC experiments at low concentrations of the hHCN4 C-linker/CNBD the protein was concentrated to 6 μM and dialyzed in 150 mM KCl, 1 mM TCEP, 30 mM HEPES, pH 7.5 overnight at 4°C using a Slide-A-Lyzer dialysis cassette (ThermoFisher Scientific). For ITC experiments at high concentrations of the hHCN4 C-linker/CNBD the protein was concentrated to 80 μM and dialyzed in 100 mM KCl, 1 mM TCEP, 20 mM HEPES, pH 7.0 overnight at 4°C using a Slide-A-Lyzer cassette. The protein solutions were degassed prior to each experiment. ITC titrations were performed at 25°C using the ITC200 calorimeter (Malvern Instruments Inc. Westborough, MA). The proteins were titrated in the 200 μL cell with 300 μM cAMP or 300 μM c-di-GMP for 6 μM C-linker/CNBDs, and with 1mM cAMP or 1 mM c-di-GMP for 80 μM C-linker/CNBDs. To test binding of c-di-GMP in the presence of cAMP with ITC, 100 μM cAMP for 6 μM C-linker/CNBDs and 1 mM cAMP for 80 μM C-linker/CNBDs were present in both the solution with hHCN4 C-linker/CNBDs and the titration solution with 300 μM c-di-GMP. The titrations were done using 1.25 μL injections at 160-s intervals. Data analysis was performed with NITPIC and SEDFAT [34,35] using a single-site binding model for 6 μM C-linker/CNBDs and a two independent binding site model for 80 μM C-linker/CNBDs, as used by Chow et al. [36].

## Results

### cAMP and cGMP bind to the immobilized C-linker/CNBD domain of mHCN2 channels in a concentration dependent manner

To test if SPR method is suitable for detecting ligand binding in HCN channels we first immobilized the isolated C-linker/CNBDs of mHCN2 channels on the NTA sensor chip using Ni^2+^-NTA and 6-His tag coupling. A schematic of the HCN2 C-linker/CNBD immobilized on the NTA sensor chip is shown in Fig 1B. We recorded SPR sensorgrams over a range of cAMP and cGMP concentrations (Fig 1C and 1D). Both cAMP and cGMP showed binding and increased the binding response in a concentration dependent manner (Fig 1C and 1D). To determine the cAMP and cGMP binding affinity the binding response was plotted against the analyte concentration (Fig 1E). Fitting of the dose response plots in Fig 1E revealed the binding affinities of 2.5 ± 0.4 μM for cAMP and 12.3 ± 1.1 μM for cGMP, The averaged binding affinities for experiments on different NTA sensor chips were 1.9 ± 0.1 μM (n = 5) for cAMP and 13.4 ± 2.0 μM (n = 7) for cGMP (Table 1). These affinities are less than the affinities determined for the full-length HCN2 channels with electrophysiology (60–500 nM for cAMP [3,37] and ~6 μM for cGMP [3]).

**Table 1:** Cyclic nucleotide binding affinities for the C-linker/CNBDs of HCN channels determined with SPR.

	K_d_ for cAMP (μM)	K_d_ for cGMP (μM)
mHCN2	1.9 ± 0.1 (n = 5)	13.4 ± 2.0 (n = 7)
mHCN2-L586W	10 ± 0.3 (n = 6)	≥ 73.9 ± 7.8 (n = 6)
hHCN4	1.5 ± 0.2 (n = 5)	5.5 ± 0.9 (n = 4)

n is the number of different Ni^2+^-NTA chips used to obtain the averaged binding affinities.

The cAMP binding affinity determined with SPR is in agreement with previously reported affinities for the isolated C-linker/CNBDs of HCN2 channels of 1–3.7 μM for cAMP determined with isothermal titration calorimetry (ITC) [13,14,36,38], lower than the binding affinity of 0.3–0.7 μM for 8-Fluo-cAMP, a fluorescent cAMP analog, determined with fluorescence anisotropy (FA) [13,14,39], and higher than the binding affinity of 10 μM for 8-AHA-cAMP, a cAMP analog, determined with SPR [13]. The differences between the affinities determined with ITC and FA reflect intrinsic differences between the two assays, as the binding affinities of 8-Fluo-cAMP and cAMP determined with ITC were similar [13,14]. To determine the binding affinity of 8-AHA-cAMP with SPR, 8-AHA-cAMP was covalently coupled to the CM5 sensor chip surface and the C-linker/CNBDs were injected at increasing concentrations [13,40]. No sensorgrams or fits of the dose-response plots for the 8-AHA-cAMP binding were included in the paper [13], precluding the direct comparison of the data with our results. The difference in the binding affinities measured with SPR for 8-AHA-cAMP and cAMP could reflect the difference in the binding affinities of cAMP and its analog, and/or the difference in the immobilization procedure used in the two studies. Importantly, the immobilization procedure used in our study is more advantageous for identification of novel HCN channel ligands using high-throughput chemical library screening than the procedure used by Lolicato et al. and Moller et al. In our study the C-linker/CNBD is immobilized on the surface and the chemical library compounds can be injected over the immobilized protein in a high-throughput manner. While in the studies by Lolicato et al. and Moller et al., a specific ligand (8-AHA-cAMP) is coupled to the sensor surface and the isolated C-linker/CNBD is injected over the immobilized ligand. Therefore, the detection of novel ligands will be cumbersome as it would require a competition with the 8-AHA-cAMP for a binding to the hHCN4 for the detection and also substantially larger amounts of the protein will be required for a large-scale screening, potentially making it impractical to screen a library of chemical compounds in a high-throughput manner with this approach.

The cGMP binding affinity determined with SPR is in agreement with previously reported affinity for the C-linker/CNBDs of HCN2 channels of 8.5 μM determined with ITC [41]. For both cAMP and cGMP two affinities, low and high, were detected with ITC [36,41]. The high affinity binding was observed at high concentrations of the protein at which the C-linker/CNBDs form tetramers. Only the low affinity binding was observed at ≤ 25 μM concentrations at which the C-linker/CNBDs are predominantly monomers. Consistent with these findings we observed only low affinity binding, as the C-linker/CNBDs are expected to be in a monomeric state at the low concentrations used for the SPR based experiments. Taken together, our results indicate that the SPR-based experimental approach described here is well suited for the detection of cyclic nucleotide binding to the isolated C-linker/CNBDs of HCN2 channels and the affinities determined with SPR are overall in agreement with affinities determined with other methods for the isolated C-linker/CNBDs.

### L586W mutation in the P-helix decreases cyclic nucleotide affinity to the immobilized C-linker/CNBDs of HCN2 channels

**Fig 2:**
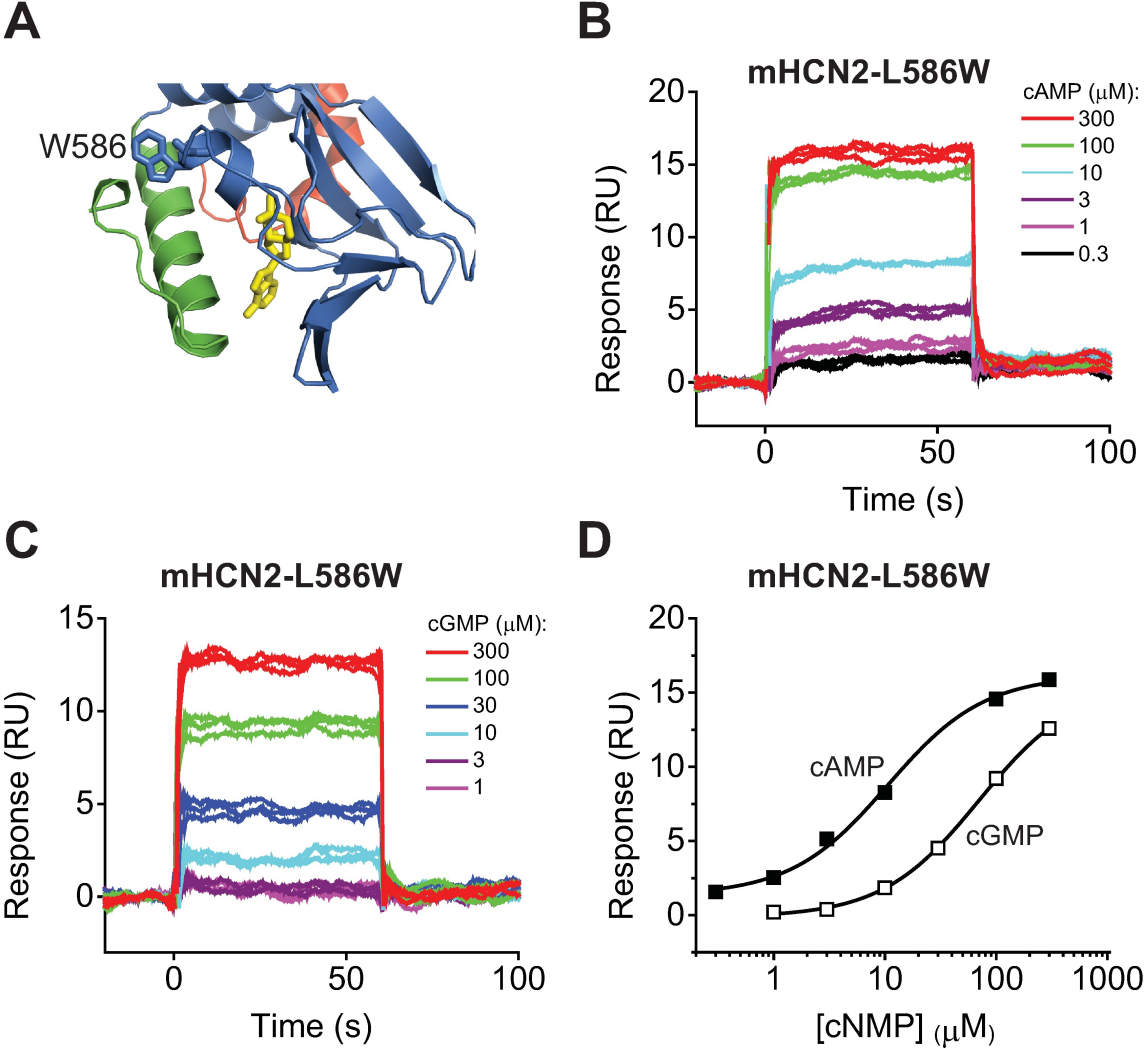
L586W mutation decreases cyclic nucleotide affinity to the C-linker/CNBDs of HCN2 channels. (A) Enlarged view of the cAMP binding pocket and the Trp residue introduced at position 586. The ribbon representation for mHCN2-L586W was obtained with SWISS-MODEL [57] based on the crystal structure of the mHCN2 C-linker/CNBD [15]. cAMP is colored in yellow. (B) and (C) Representative SPR sensorgrams recorded for the immobilized HCN2-L586W C-linker/CNBDs with the indicated concentrations of cAMP (B) and cGMP (C). C-linker/CNBDs immobilized on the same surface were used in (B) and (C). (D) Plots of the SPR response at 30 s after the start of the injection versus total cAMP (filled squares) and cGMP (open squares) concentration for sensorgrams shown in (B) and (C). The lines represent fits of the data with Hill equation. The binding affinities were 10.6 ± 1.5 μM for cAMP and ≥70.1 ± 3.8 μM for cGMP.

Previously, mHCN2-L586W C-linker/CNBDs were used to determine cAMP and cGMP binding affinities using the tryptophan fluorescence as a reporter of the cyclic nucleotide binding [27]. The C-linker/CNBDs of mHCN2 channels lack endogenous tryptophan residues. Therefore, a tryptophan residue was substituted for the leucine at the position 586 on the P-helix near the cyclic nucleotide binding site (Fig 2A). Cyclic nucleotide binding affinities measured based on the changes in the tryptophan fluorescence were 13 ± 2 μM for cAMP and 62 ± 23 μM for cGMP [27]. These affinities are lower than the affinities determined with SPR. To distinguish if the discrepancy in the affinities is due to the intrinsic differences between the two assays used or due to the changes in the affinity introduced by the L586W mutation, we immobilized the mHCN2-L586W C-linker/CNBDs on the NTA sensor chip and recorded SPR sensorgrams over a range of cAMP and cGMP concentrations (Fig 2B and 2C). Fitting of the dose response plots revealed the binding affinities of 10.6 ± 1.5 μM for cAMP and ≥70.1 ± 3.8 μM for cGMP (Fig 2D), and averaged binding affinities of 10.0 ± 0.3 μM (n = 6) for cAMP and >73.9 ± 7.8 μM (n = 6) for cGMP for experiments on different NTA sensor chips (Table 1). At concentrations higher than 300 μM cyclic nucleotides bound strongly to the reference surface with no immobilized protein. Therefore, the response at these high concentrations had to be excluded from the affinity calculations. Because of this limitation we were able to estimate only the lower limit of cGMP affinity.

The cAMP affinity for the L586W mutant C-linker/CNBDs determined with SPR was comparable to the affinity for the mutant domains determined with the tryptophan fluorescence-based method and lower than the affinity for the wild-type domains determined with SPR (Table 1). Although we were unable to determine the exact cGMP affinity for the L586W mutant C-linker/CNBDs, it is much lower than the affinity for the wild-type domains (Table 1). These observations indicate the L586W mutation decreases the cyclic nucleotide affinity for the C-linker/CNBDs of HCN2 channels and the two assays, SPR and tryptophan fluorescence-based, give similar measurements for cyclic nucleotide binding affinities.

### cAMP and cGMP bind to the immobilized C-linker/CNBD of hHCN4 channels in a concentration dependent manner

**Fig 3:**
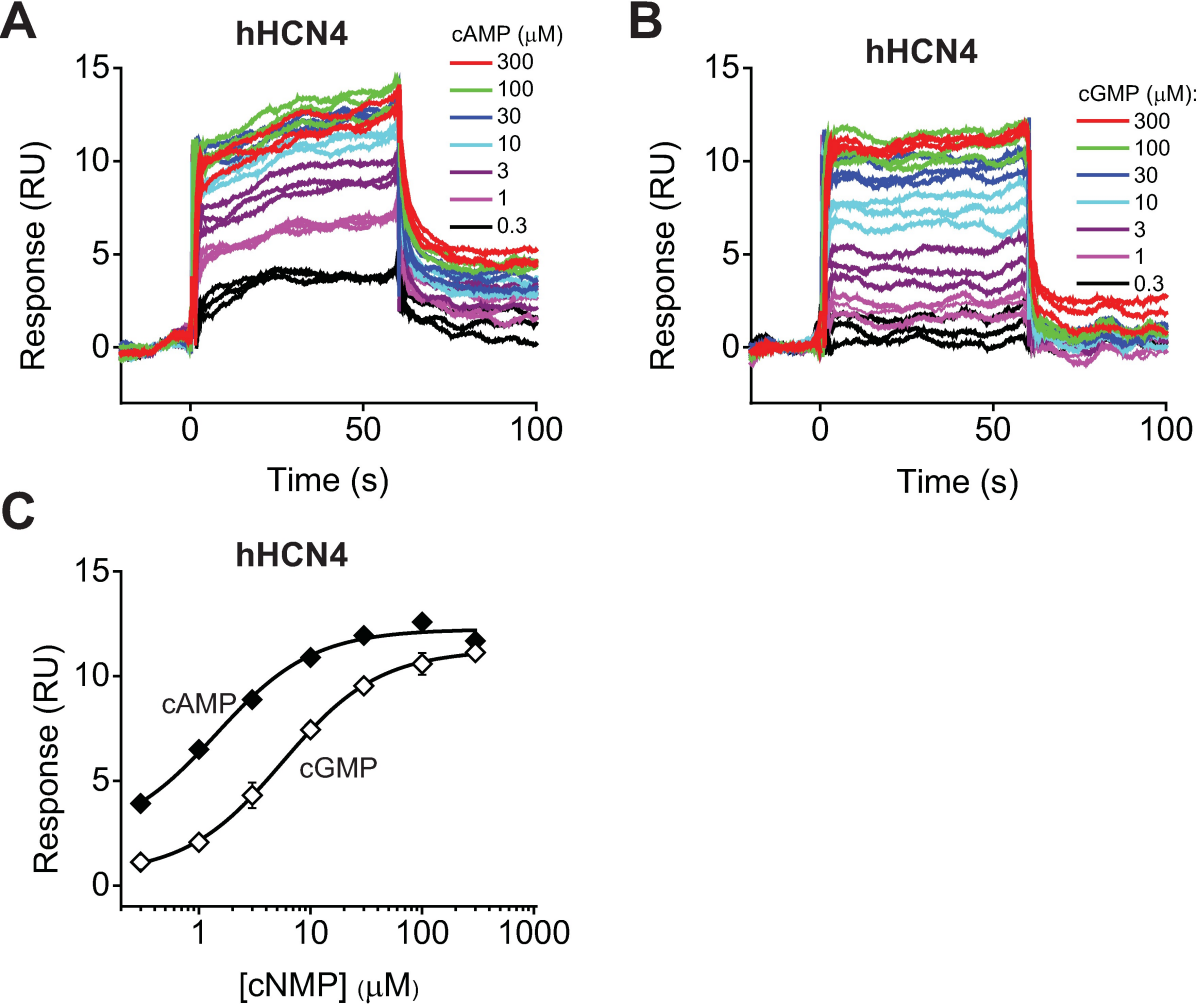
Detecting cNMP binding to the HCN4 C-linker/CNBDs with SPR. (A) and (B) Representative SPR sensorgrams recorded for the immobilized hHCN4 C-linker/CNBDs with the indicated concentrations of cAMP (A) and cGMP (B). C-linker/CNBDs immobilized on the same surface were used in (A) and (B). (C) Plots of the SPR response at 30 s after the start of the injection versus total cAMP (filled diamonds) and cGMP (open diamonds) concentration for sensorgrams shown in (A) and (B). The lines represent fits of the data with Hill equation. The binding affinities were 1.4 ± 0.3 μM for cAMP and 5.6 ± 0.2 μM for cGMP.

To determine the cyclic nucleotide affinity for the C-linker/CNBDs of HCN4 channels with SPR, we immobilized the isolated C-linker/CNBDs of hHCN4 channels on the NTA sensor chip and recorded SPR sensorgrams over a range of cAMP and cGMP concentrations (Fig 3A and 3B). Both cAMP and cGMP increased the binding response in a concentration dependent manner (Fig 3A and 3B). The analysis of the dose response plots revealed binding affinities of 1.4 ± 0.3 μM for cAMP and 5.6 ± 0.2 μM for cGMP (Fig 3C), and averaged binding affinities of 1.5 ± 0.2 μM (n = 5) for cAMP and 5.5 ± 0.9 μM (n = 4) for cGMP for experiments on different NTA sensor chips (Table 1). The cAMP affinity for the C-linker/CNBDs of HCN4 was similar to the affinity for the C-linker/CNBDs of HCN2 channels (P > 0.1 by ANOVA), while the cGMP affinity was about two-fold higher for hHCN4 than for mHCN2 domains (P < 0.05 by ANOVA). The cAMP binding affinity for the isolated HCN4 C-linker/CNBDs was the same as the cAMP affinity of 1.5 μM reported for the full-length hHCN4 channels with electrophysiology [42] and cGMP affinity was higher for the isolated domains than the affinity of 13.2 μM determined with electrophysiology [23].

The cAMP binding affinity for the isolated C-linker/CNBDs of HCN4 channels determined with SPR is lower than the previously reported binding affinity for the isolated domains of ~ 0.8 μM for cAMP determined with ITC [14,36] and of 0.18–0.28 μM for 8-Fluo-cAMP determined with FA [13,14], and is in the lower end of the 1–9 μM range for cAMP affinities determined with the Saturation Transfer Reference method [21]. The cAMP affinity determined here is higher than the affinity of 11 μM for 8-AHA-cAMP binding determined with SPR using 8-AHA-cAMP covalently coupled to the CM5 sensor [13]. The cGMP affinity determined here is lower than the 8-Fluo-cGMP affinity of 0.7 μM for the C-linker/CNBDs of HCN4 channels determined with FA [13]. As mentioned above the differences in the cyclic nucleotide affinities determined with different methods could reflect the intrinsic differences in the assays used and also the differences in the binding affinities of various cyclic nucleotide analogs. Our results indicate that similar to the measurements of cyclic nucleotide affinity to the HCN2 C-linker/CNBDs, the SPR-based method can be used to measure cyclic nucleotide affinity to the isolated domains of HCN4 channels.

### Cyclic dinucleotides do not bind to the immobilized C-linker/CNBD domain of hHCN4 channels

**Fig 4:**
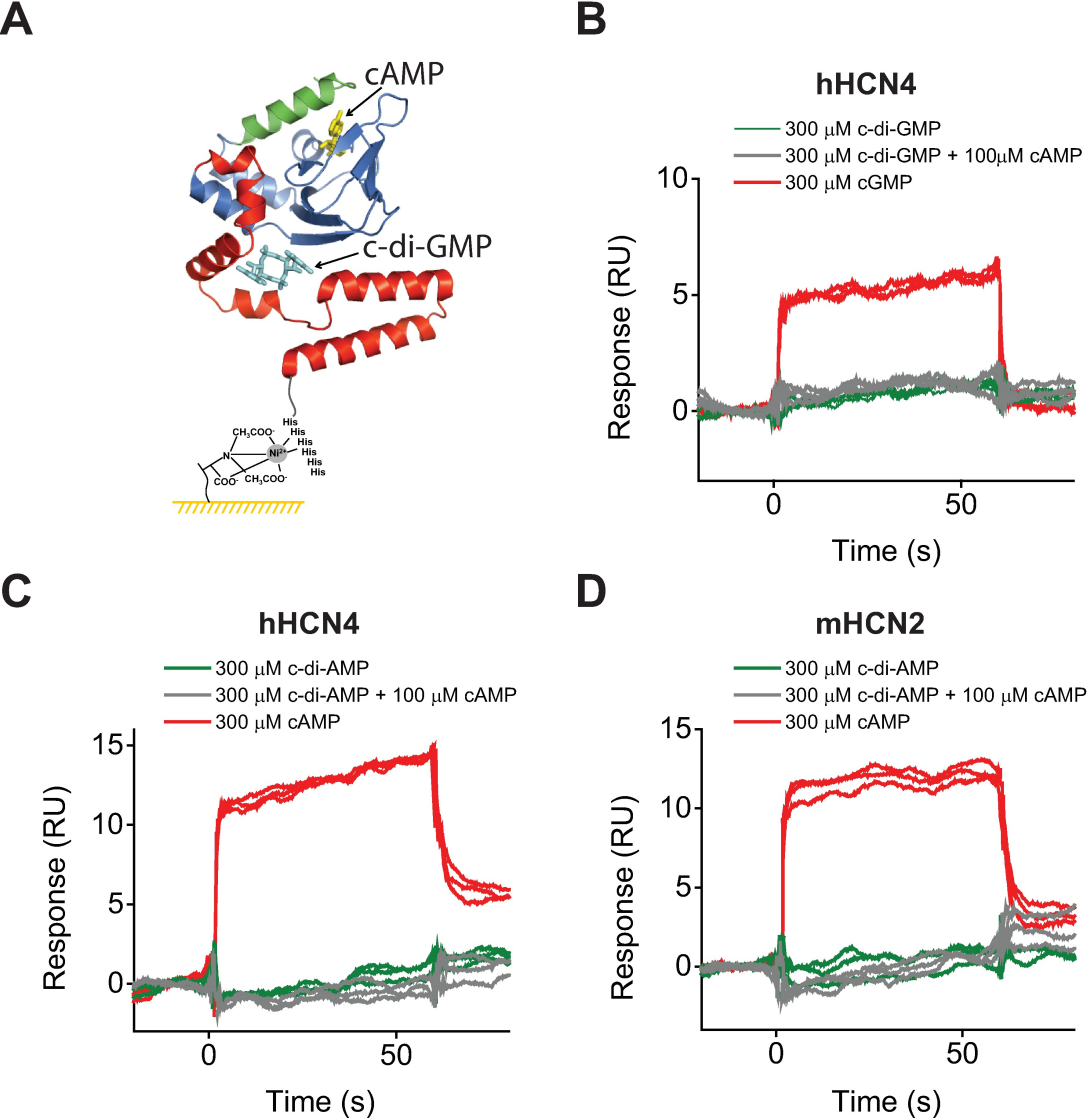
Cyclic dinucleotides do not bind to the HCN4 and HCN2 C-linker/CNBDs. (A) Cartoon of the hHCN4 C-linker/CNBD immobilized on the NTA chip surface using Ni^2+^-NTA and the N-terminal 6-His tag coupling. The same color coding as in Fig 1B. c-di-GMP placed in the proposed CLP site is shown in cyan. The ribbon representation of the C-linker/CNBD is according to ref [14]. (B) Representative SPR sensorgrams recorded for the immobilized hHCN4 C-linker/CNBDs in the presence of 300 μM c-di-GMP (green), 300 μM c-di-GMP and 100 μM cAMP (grey), and 300 μM cGMP (red). All analytes were injected over the same surface with immobilized hHCN4 C-linker/CNBDs. No increase in the binding response was detected upon injection of c-di-GMP in the presence or absence of cAMP (grey and green traces). (C) Representative SPR sensorgrams recorded for the immobilized hHCN4 C-linker/CNBDs in the presence of 300 μM c-di-AMP (green), 300 μM c-di-AMP and 100 μM cAMP (grey), and 300 μM cAMP (red). No increase in the binding response was detected upon injection of c-di-AMP in the presence or absence of cAMP (grey and green traces). All analytes were injected over the same surface with immobilized hHCN4 C-linker/CNBDs. (D) Representative SPR sensorgrams recorded for the immobilized mHCN2 C-linker/CNBDs in the presence of 300 μM c-di-AMP (green), 300 μM c-di-AMP and 100 μM cAMP (grey), and 300 μM cAMP (red). No increase in the binding response was detected upon injection of c-di-AMP in the presence or absence of cAMP (grey and green traces). All analytes were injected over the same surface with immobilized mHCN2 C-linker/CNBDs.

It was shown that in the presence of cyclic nucleotides, cyclic dinucleotides, including c-di-GMP, c-di-AMP and cGAMP, antagonized the effect of cyclic nucleotides [23]. For instance, c-di-GMP decreased the positive shift in the hHCN4 half-maximal activation voltage (V_1/2_) of 17 mV induced by 15 μM cAMP in a concentration dependent manner with an apparent affinity of 1.8 μM [23]. Analysis of the HCN4 C-linker/CNBD domain structure crystallized in the presence of cGMP revealed a canonical cGMP binding site in the β-roll cavity and also a second site in the C-linker formed at the interface with the β-roll, referred as the C-linker pocket (CLP), as illustrated in Figs 1A and 4A. The CLP site was big enough to accommodate cyclic dinucleotides and structure-based molecular docking simulations placed cyclic dinucleotides inside the CLP site [23]. Mutations of the C-linker residues Y559, F564 and E566, predicted to form direct contacts with cyclic dinucleotides by the molecular docking simulations, affected cyclic dinucleotide modulation and R680E mutation in the β8 strand of the β-roll completely abolished the effect of cyclic dinucleotides, while having little effect on the cyclic nucleotide induced shift in the V_1/2_ [23]. Although direct evidence for cyclic dinucleotide binding was missing, based on the structural and mutational analysis it was proposed that cyclic dinucleotides modulate hHCN4 channels via direct binding to the C-linker/CNBDs.

To test if cyclic dinucleotides directly bind to the isolated C-linker/CNBDs of hHCN4 channels we immobilized the isolated domains on the NTA sensor chip (Fig 4A) and recorded SPR sensorgrams in the presence of cyclic dinucleotides. No binding to the immobilized C-linker-CNBDs was detected for c-di-GMP at 300 μM concentration (Fig 4B, green traces). The cyclic dinucleotide effect on the full-length hHCN4 channels was detected only in the presence of cyclic nucleotides, with 100 μM c-di-GMP completely abolishing the effect of 15 μM cAMP on the V_1/2_ [23]. Therefore, to test if the cyclic dinucleotide binding to the immobilized C-linker/CNBDs requires cyclic nucleotides we recorded SPR sensorgrams in the presence of both c-di-GMP and cAMP. For these experiments the blank subtraction was carried out as described in “Experimental Procedures” but using the buffer with 100 μM cAMP as a blank. In the presence of 100 μM cAMP no increase in the binding response was detected upon injection of 300 μM c-di-GMP (Fig 4B, grey traces). As a positive control we injected 300 μM cGMP alone (Fig 4B, red traces) and recorded a robust response (Fig 4B, red traces). All three triplicate injections (300 μM c-di-GMP, 300 μM c-di-GMP + 100 μM cAMP, and 300 μM cGMP) were carried out over the same immobilized hHCN4 C-linker/CNB domains. The use of the same chip surface for the three injections removes the possibility of no binding detection due to an improper C-linker/CNBDs immobilization on the NTA sensor chip.

No binding was also observed for c-di-AMP injected at 300 μM concentrations to the immobilized hHCN4 C-linker/CNBDs in the absence (Fig 4C, green traces) or presence of 100 μM cAMP (Fig 4C, grey traces), while a robust binding was detected for 300 μM cAMP alone (Fig 4C, red traces). All three injections were also carried out on the same immobilized C-linker/CNBDs. The SPR sensorgrams recorded over the range of c-di-GMP, c-di-AMP and cGAMP in the absence and presence of 100 μM cAMP also did not show any cyclic dinucleotide binding (S2 Fig). In agreement with the report that HCN2 channels are not regulated by cyclic dinucleotides [23], C-linker/CNBDs of HCN2 channels did not show any cyclic dinucleotide binding in the absence or presence of cAMP when measured with SPR, while a robust binding for cAMP alone was recorded (Fig 4D). These results indicate that cyclic dinucleotides do not bind to the immobilized isolated C-linker/CNBDs of HCN4 and HCN2 channels.

### Cyclic dinucleotides do not bind to the non-immobilized monomeric and tetrameric C-linker/CNBD domain of hHCN4 channels

**Fig 5:**
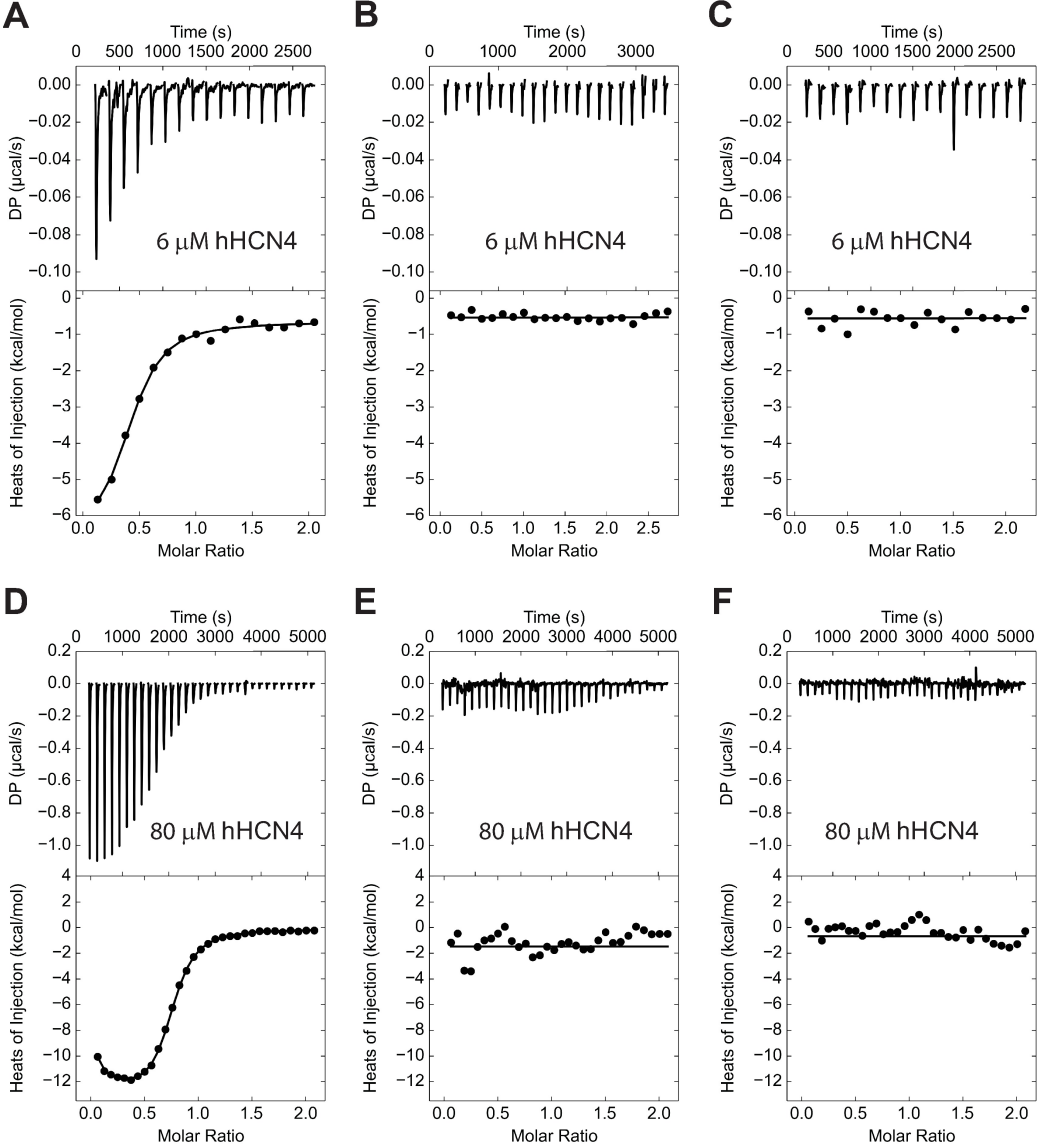
Cyclic nucleotide and cyclic dinucleotide binding to the monomeric and tetrameric HCN4 C-linker/CNBD tested with ITC. Thermograms of successive injections of 1.25 μl of cAMP (A and D), c-di-GMP (B and E) and c-di-GMP in the presence of cAMP (C and F) (top panels) and the corresponding binding isotherms (bottom panels). For experiments in (C) 100 μM cAMP and in (F) 1 mM cAMP was present in both the protein and ligand solutions. Monomeric 6-His tagged hHCN4 C-linker/CNBDs at 6 μM concentration, purified in the same manner as for the SPR-based experiments, were used for experiments in (A-C). hHCN4 C-linker/CNBDs after the MBP tag cleavage at 80 μM concentration were used for experiments in (D-F). The binding isotherms were obtained by integrating the peaks in the top panels, normalizing the obtained values by the cAMP concentration and plotting them against the molar ratio of cAMP to the protein. The lines represent a nonlinear least-square fit to a single–site binding model for (A-C) and a two independent binding site model for (D-F). The binding affinities for cAMP were 1.1 ± 0.5 μM in (A), and 1.7 ± 0.3 μM and 0.03 ± 0.02 μM in (D).

To test if the observed absence of cyclic dinucleotide binding to the isolated C-linker/CNBDs of hHCN4 is due to a possible change in the C-linker conformation caused by the immobilization of the protein on the sensor chip surface we used isothermal titration calorimetry (ITC), a method that does not require protein immobilization, to determine ligand binding to the 6-His tagged C-linker/CNBDs used in the SPR experiments. With ITC experiments on the C-linker/CNBDs at 6 μM concentrations we observed cAMP binding with the binding affinity of 1.1 ± 0.5 μM (Fig 5A), in agreement with the previously reported binding affinities obtained with this method at low concentrations of the C-linker/CNBDs at which they are expected to be in a monomeric form [14,36]. However, similar to the SPR results, no binding was observed for c-di-GMP either in the absence (Fig 5B) or presence of 100 μM cAMP (Fig 5C). For the experiments in Fig 5C, 100 μM cAMP was present in both the ligand and protein solutions. Therefore, the absence of cyclic dinucleotide binding to the C-linker/CNBDs of hHCN4 observed with SPR is not due to the immobilization of the protein on the sensor chip.

In the absence of cAMP the C-linker/CNBDs of HCN4 channels are primarily monomeric while increase in the cAMP concentration and the concentration of the C-linker/CNBDs promotes formation of dimers and tetramers [13,15,36]. The effect of cyclic dinucleotides on HCN4 currents was observed only in the presence of cAMP [23]. Therefore, cyclic dinucleotide binding might require tetramerization of the C-linker/CNBDs. To test this possibility we first expressed the C-linker/CNBDs of hHCN4 fused to the maltose binding protein (MBP), since the MBP tag typically increases the yield of the expressed protein [43,44]. We then cleaved the MBP tag with thrombin, concentrated the purified hHCN4 C-linker/CNBDs at 80 μM concentration and examined ligand binding with ITC. We observed cAMP binding to the purified protein with affinities of ~ 1.7 ± 0.3 μM and 0.03 ± 0.02 μM (Fig 5D). These affinities were similar to the low- and high-binding affinities of 1.11 ± 0.62 μM and 0.07 ± 0.05 μM determined with ITC at high concentrations of hHCN4 C-linker/CNBDs by Chow et al [36]. It has been proposed that a negative cooperativity in cAMP binding to the tetrameric C-linker/CNBDs could account for the presence of high-and low-binding affinities [36]. However, similar to the ITC and SPR results at low concentration of the C-linker/CNBDs, no binding was observed for c-di-GMP either in the absence (Fig 5E) or presence of 1 mM cAMP at high concentrations of C-linker/CNBDs (Fig 5F). It has been shown with analytical ultracentrifugation and size exclusion chromatography that in the presence of 1 mM cAMP and at concentrations of ~70 uM or higher the C-linker/CNBDs of hHCN4 channels are found only as tetramers [13]. Therefore, our experiments indicate that tetrameric C-linker/CNBDs of hHCN4 channels also do not bind cyclic dinucleotides.

## Discussion

Here we show that SPR can be used to accurately measure ligand binding to the isolated C-linker/CNBDs of HCN channels. We found that binding affinity of cyclic nucleotides, cAMP and cGMP, to the C-linker-CNBDs of mHCN2 and hHCN4 channels determined with SPR is comparable to the affinities measured with other methods, indicating that SPR is well suited for studying ligand-binding in HCN channels. Unlike for cyclic nucleotides, we did not detect any binding for cyclic dinucleotides, c-diGMP, c-diAMP and cGAMP, to the C-linker-CNBDs of mHCN2 and hHCN4 channels either in the absence or presence of cyclic nucleotides when measured with SPR and also with ITC. These findings indicate that the isolated C-linker/CNBD of HCN4 channels is insufficient for direct binding of cyclic dinucleotides.

### Potential mechanisms for cyclic dinucleotides modulation of HCN channels

Cyclic dinucleotides are common signaling molecules in bacteria [24]. Although recently they have been also discovered in eukaryotes [25,26], the physiological role of cyclic dinucleotides in higher organisms is not yet clear. Cyclic dinucleotides regulate HCN channels in a subtype dependent manner [23]. They antagonized the effect of cyclic nucleotides in HCN4 channels but have no effect on HCN2 channels [23]. Although at this point cyclic dinucleotides are not regarded as physiologically relevant HCN channel ligands, understanding the mechanisms of cyclic dinucleotide regulation might facilitate the discovery of novel subtype specific regulators. Structural analysis of the C-linker region and mutagenesis studies suggested that the C-linker forms a binding site for cyclic dinucleotides in HCN4 channels, although a direct evidence of the binding was missing [23]. Interestingly, the C-linker/CNBDs of HCN4 and HCN2 channels are highly conserved in their amino acid sequence and structural fold (S1 Fig) [14,15]. Moreover, residues that had been shown to affect cyclic dinucleotide modulation of HCN4 channels, including R680 that completely abolished the cyclic dinucleotide effect, are identical in HCN2 and HCN4 channels (S1 Fig). Therefore, if the C-linker/CNBDs are solely responsible for the cyclic dinucleotide binding, it is difficult to reconcile the differential modulation of HCN4 and HCN2 channels by cyclic dinucleotides. Our observation that the isolated monomeric and tetrameric C-linker/CNBDs of HCN4 channels do not bind cyclic dinucleotides might provide the answer to this conundrum, as it suggests that to bind cyclic dinucleotides the C-linker might require interaction with other regions of the channel.

**Fig 6:**
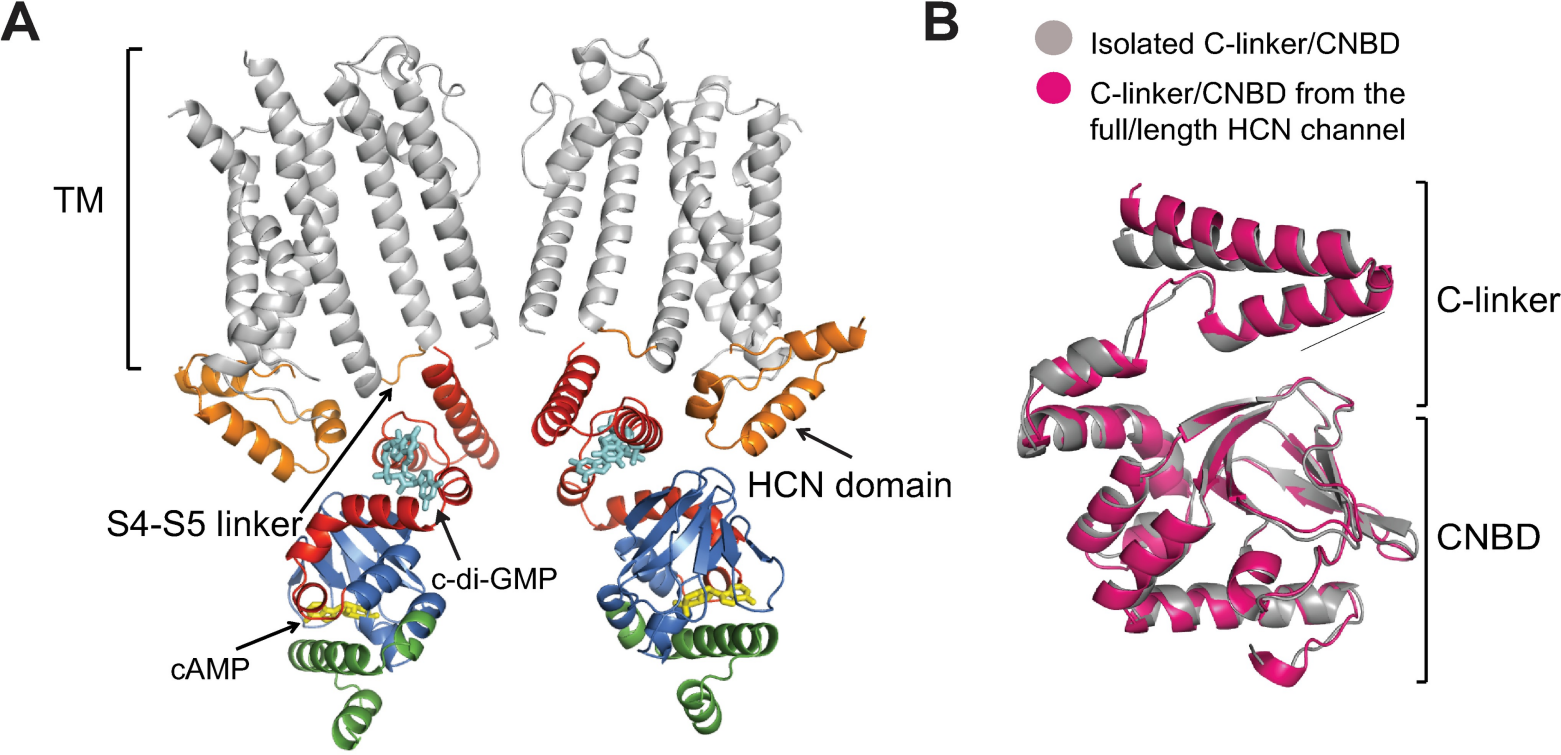
Structural model of the c-di-GMP regulation. (A) Ribbon representation of transmembrane segments of two opposing subunits and the C-linker/CNBDs of the two subunits adjacent to them from the full-length structure of HCN1 channels [45]. The transmembrane segments of the subunits containing the C-linker/CNBDs shown in the figure and the C-linker/CNBDs of the subunits containing transmembrane segments shown in the figure are omitted for clarity. In the crystal structure C-linkers are making direct contacts with S4-S5 linkers and HCN domains from only the adjacent subunits. Transmembrane segments (TM) are shown in grey, S4-S5 linkers and HCN domains are orange, C-linkers are red, β-roll and helices A, P and B are blue, and the C-helix and the distal C-terminus are green. cAMP bound inside the β-roll cavity is yellow. c-di-GMP placed in the proposed CLP site is cyan. (B) Structural alignment of the C-linker/CNBD from the full-length HCN1 structure [45] shown in gray and isolated C-linker/CNBD of HCN2 channels [15] shown in magenta.

What are the regions of HCN channels that may facilitate the cyclic dinucleotide binding to the C-linker and give rise to the differential regulation of HCN4 and HCN2 channels? Recent cryo-electron microscopy structure of the full-length HCN1 channel [45] and functional studies [46] indicate that the S4-S5 linkers directly interact with the C-linkers from neighboring subunits in HCN channels (Fig 6A). However, the S4-S5 linkers are conserved in HCN2 and HCN4 channels, making this interaction an unlikely source of the differential effect of cyclic dinucleotides. Another region revealed by the crystal structure to form direct interactions with the C-linker is the HCN domain, comprised of three alpha helices directly preceding the first transmembrane segment in HCN channels (Fig 6A) [45]. The HCN domain might be sufficiently diverse to give rise to the subtype specific effect of cyclic dinucleotides on HCN channel function. Alternatively, subtle structural differences between the full-length HCN2 and HCN4 channels may be responsible for the differential modulation by cyclic dinucleotides.

Structural alignment of the C-linker/CNBDs from the cryo-electron microscopy structure of HCN1 channels [45] with the crystal structure of the isolated C-linker/CNBD of HCN2 channels [15] revealed a high similarity with the root mean square deviation (r.m.s.d.) of 1 Å (Fig 6B). Remarkably the fold and orientation of the C-linker in the isolated C-linker/CNBD, which is not coupled to the S6 transmembrane segment, is very similar to the orientation of the C-linker in the full length structure. This similarity underscores the relevance of the findings from the studies on the isolated C-linker/CNBDs for understanding the molecular mechanisms of the full-length HCN channel function.

Beyond the considered molecular mechanisms membrane-associated factors, posttranslational modifications and interacting proteins could also contribute to the isoform-specific cyclic dinucleotide modulation of HCN channels. Since the effect of cyclic dinucleotides was preserved for HCN channels in excised inside-out patches [23], these mechanisms have to be membrane-delimited. For instance, it has been shown that in Chinese hamster ovary (CHO) cells external application of cAMP did not increase HCN4 channel activity in both whole-cell recordings and excised patches as the basal voltage dependence was already shifted to more depolarized potentials [47]. This tonic activation of HCN4 channels was absent for HCN2 channels, and was attributed to the distal C terminus of HCN4 channels (downstream of the CNBD) and an unidentified membrane-associated intracellular factor(s) specific to CHO cells. The distal C-terminus is diverse between HCN2 and HCN4 channels and could promote isoform-specific regulation. Therefore, it is conceivable that a membrane-associated intracellular factor, which would be missing in our experiments based on the purified C-linker/CNBDs, is contributing to cyclic dinucleotide binding and modulation in HCN4 channels.

HCN channels contain multiple consensus PKA (cAMP-activated protein kinase) phosphorylation sites and display isoform and cell-type specific PKA-dependent regulation [48]. PKA was shown to regulate the activity of native HCN channels in mouse sinoatrial myocytes [48] and rat olfactory receptor neurons [49], and HCN4 channels heterologously expressed in CHO cells via a direct phosphorylation [48]. However, PKA had no effect on native HCN channels in rat dorsal root ganglion cells [50] and guinea pig sensory afferent neurons [51]. Interestingly, the diverse distal C-terminus was required for the effect of PKA on HCN4 channels [48]. Therefore, it is possible that the isoform-dependent phosphorylation state could contribute to the isoform-specific cyclic dinucleotide modulation of HCN channels.

Finally, membrane-associated proteins interacting with HCN channels can also play a role in cyclic dinucleotide modulation. One of the known interacting proteins for HCN channels is tetratricopeptide repeat-containing Rab8b-interacting protein (TRIP8b), an auxiliary cytoplasmic protein [52,53]. TRIP8b antagonizes the effect of cAMP by directly interacting with the C-linker/CNBD of HCN channels [54,19]. As a cytoplasmic protein that affects both HCN2 and HCN4 channels to the same extend, TRIP8b is unlikely to contribute to the isoform specificity of the cyclic dinucleotide modulation seen in excised patches. However, it is possible that yet unidentified membrane-associated protein could be affecting cyclic dinucleotide modulation of HCN channels.

### Potential of SPR for HCN channel drug discovery

SPR is a powerful biophysical method for quantitatively investigating ligand-protein interactions [55,56]. It uses changes in the refractive index of the material near the sensor surface with immobilized receptor to detect ligand binding. The SPR method has several advantages for studies of ligand binding: 1) The detection of ligand binding with the SPR approach used in our study does not require development of a radioactive or fluorescent ligand analogs. This makes it possible to detect unknown ligands via screening of chemical libraries, a collection of thousands of small molecules that could be potential novel ligands, for binding to the target protein; 2) SPR offers an excellent opportunity of a direct comparison of affinities for various ligands as they can be measured for the same immobilized receptor. Here we used this advantage to probe cyclic nucleotide and cyclic dinucleotide binding to the same C-linker/CNBDs (Fig 4); 3) SPR requires small amounts of protein for immobilization on the chip surface and once immobilized the same surface can be used for the dose-response experiments for several different ligands; 4) Finally, SPR offers a great platform for high-throughput screening of chemical libraries with a capacity of detecting the binding of up to 4800 compounds in 24 hrs. It should be noted that the SPR method also has a limitation. It requires protein immobilization on the sensor chip surface, which could affect the protein conformation and, therefore, ligand binding. However, we feel that the advantages of the method far outweigh the limitation of the SPR for ion channel drug discovery.

HCN channels are important drug discovery targets for cardiovascular and neurological disorders. There is a need for the development of a robust high-throughput screening platform for the discovery of novel HCN channel small molecule regulators. Our study indicates that SPR is well suited for ligand binding studies in HCN channels and paves the way for the discovery of novel HCN channel regulators using SPR as a high-throughput chemical library screening platform.

## Supporting information

S1 Fig Amino acid sequence and structural alignment of the C-linker/CNBDs of mHCN2 and hHCN4. Identical residues are black on yellow background, similar residues are black on green background. The residues mutations of which altered cyclic dinucleotide response are indicated by arrows. Protein accession numbers are EDL31671 for mHCN2 and Q9Y3Q4 for hHCN4. (B) Structural alignment of the C-linker/CNBD of mHCN2 [15] (gray) and hHCN4 channels [14] (blue). cAMP bound inside the β-roll cavity is yellow and c-di-GMP placed in the proposed CLP site is cyan.
(PDF)

S2 Fig Cyclic dinucleotides do not bind to the HCN4 C-linker/CNBDs. Representative SPR sensorgrams recorded for the immobilized hHCN4 C-linker/CNBDs in the absence (A, C, E) and presence (B, D, F) of 100 μM cAMP with the indicated concentrations of c-di-GMP (A and B), c-di-AMP (C and D) and cGAMP (E and F). No increase in the binding response was detected upon injection of cyclic dinucleotides at the indicated concentration in the absence or presence of 100 μM cAMP.
(PDF)

## References

[1] Robinson RB, Siegelbaum SA. Hyperpolarization-activated cation currents: from molecules to physiological function. Annu Rev Physiol. 2003;65:453–80. doi: 10.1146/annurev.physiol.65.092101.142734 12471170 10.1146/annurev.physiol.65.092101.142734

[2] Santoro B, Grant SG, Bartsch D, Kandel ER. Interactive cloning with the SH3 domain of N-src identifies a new brain specific ion channel protein, with homology to eag and cyclic nucleotide-gated channels. Proc Natl Acad Sci U S A. 1997;94(26):14815–20. 9405696 10.1073/pnas.94.26.14815 PMC25120

[3] Ludwig A, Zong X, Jeglitsch M, Hofmann F, Biel M. A family of hyperpolarization-activated mammalian cation channels. Nature. 1998;393(6685):587–91. doi: 10.1038/31255 9634236 10.1038/31255

[4] Santoro B, Liu DT, Yao H, Bartsch D, Kandel ER, Siegelbaum SA, et al Identification of a gene encoding a hyperpolarization-activated pacemaker channel of brain. Cell. 1998;93(5):717–29. 9630217 10.1016/s0092-8674(00)81434-8

[5] Santoro B, Chen S, Luthi A, Pavlidis P, Shumyatsky GP, Tibbs GR, et al Molecular and functional heterogeneity of hyperpolarization-activated pacemaker channels in the mouse CNS. J Neurosci. 2000;20(14):5264–75. 10884310 10.1523/JNEUROSCI.20-14-05264.2000 PMC6772310

[6] Moosmang S, Biel M, Hofmann F, Ludwig A. Differential distribution of four hyperpolarization-activated cation channels in mouse brain. Biol Chem. 1999;380(7–8):975–80. doi: 10.1515/BC.1999.121 10494850 10.1515/BC.1999.121

[7] Monteggia LM, Eisch AJ, Tang MD, Kaczmarek LK, Nestler EJ. Cloning and localization of the hyperpolarization-activated cyclic nucleotide-gated channel family in rat brain. Brain Res Mol Brain Res. 2000;81(1–2):129–39. 11000485 10.1016/s0169-328x(00)00155-8

[8] Moosmang S, Stieber J, Zong X, Biel M, Hofmann F, Ludwig A. Cellular expression and functional characterization of four hyperpolarization-activated pacemaker channels in cardiac and neuronal tissues. Eur J Biochem. 2001;268(6):1646–52. 11248683 10.1046/j.1432-1327.2001.02036.x

[9] Ishii TM, Takano M, Xie LH, Noma A, Ohmori H. Molecular characterization of the hyperpolarization-activated cation channel in rabbit heart sinoatrial node. J Biol Chem. 1999;274(18):12835–9. 10212270 10.1074/jbc.274.18.12835

[10] Shi W, Wymore R, Yu H, Wu J, Wymore RT, Pan Z, et al Distribution and prevalence of hyperpolarization-activated cation channel (HCN) mRNA expression in cardiac tissues. Circ Res. 1999;85(1):e1–e6. 10400919 10.1161/01.res.85.1.e1

[11] DiFrancesco D. Pacemaker mechanisms in cardiac tissue. Annu Rev Physiol. 1993;55:455–72. doi: 10.1146/annurev.ph.55.030193.002323 7682045 10.1146/annurev.ph.55.030193.002323

[12] Pape HC, McCormick DA. Noradrenaline and serotonin selectively modulate thalamic burst firing by enhancing a hyperpolarization-activated cation current. Nature. 1989;340(6236):715–8. doi: 10.1038/340715a0 2475782 10.1038/340715a0

[13] Lolicato M, Nardini M, Gazzarrini S, Moller S, Bertinetti D, Herberg FW, et al Tetramerization dynamics of C-terminal domain underlies isoform-specific cAMP gating in hyperpolarization-activated cyclic nucleotide-gated channels. J Biol Chem. 2011;286(52):44811–20. doi: 10.1074/jbc.M111.297606 22006928 10.1074/jbc.M111.297606 PMC3247997

[14] Xu X, Vysotskaya ZV, Liu Q, Zhou L. Structural basis for the cAMP-dependent gating in the human HCN4 channel. J Biol Chem. 2010;285(47):37082–91. doi: 10.1074/jbc.M110.152033 20829353 10.1074/jbc.M110.152033 PMC2978636

[15] Zagotta WN, Olivier NB, Black KD, Young EC, Olson R, Gouaux E. Structural basis for modulation and agonist specificity of HCN pacemaker channels. Nature. 2003;425(6954):200–5. doi: 10.1038/nature01922 12968185 10.1038/nature01922

[16] Taraska JW, Puljung MC, Zagotta WN. Short-distance probes for protein backbone structure based on energy transfer between bimane and transition metal ions. Proc Natl Acad Sci U S A. 2009;106(38):16227–32. Epub 2009/10/07. doi: 10.1073/pnas.0905207106 ; PubMed Central PMCID: PMCPMC2741476. 19805285 10.1073/pnas.0905207106 PMC2741476

[17] Puljung MC, DeBerg HA, Zagotta WN, Stoll S. Double electron-electron resonance reveals cAMP-induced conformational change in HCN channels. Proc Natl Acad Sci U S A. 2014;111(27):9816–21. Epub 2014/06/25. doi: 10.1073/pnas.1405371111 ; PubMed Central PMCID: PMCPMC4103371. 24958877 10.1073/pnas.1405371111 PMC4103371

[18] Zhou L, Siegelbaum SA. Gating of HCN channels by cyclic nucleotides: residue contacts that underlie ligand binding, selectivity, and efficacy. Structure. 2007;15(6):655–70. Epub 2007/06/15. doi: 10.1016/j.str.2007.04.012 ; PubMed Central PMCID: PMCPMC1995447. 17562313 10.1016/j.str.2007.04.012 PMC1995447

[19] Saponaro A, Pauleta SR, Cantini F, Matzapetakis M, Hammann C, Donadoni C, et al Structural basis for the mutual antagonism of cAMP and TRIP8b in regulating HCN channel function. Proc Natl Acad Sci U S A. 2014;111(40):14577–82. doi: 10.1073/pnas.1410389111 25197093 10.1073/pnas.1410389111 PMC4210022

[20] Goldschen-Ohm MP, Klenchin VA, White DS, Cowgill JB, Cui Q, Goldsmith RH, et al Structure and dynamics underlying elementary ligand binding events in human pacemaking channels. Elife. 2016;5 doi: 10.7554/eLife.20797 27858593 10.7554/eLife.20797 PMC5115869

[21] Akimoto M, Zhang Z, Boulton S, Selvaratnam R, VanSchouwen B, Gloyd M, et al A mechanism for the auto-inhibition of hyperpolarization-activated cyclic nucleotide-gated (HCN) channel opening and its relief by cAMP. J Biol Chem. 2014;289(32):22205–20. doi: 10.1074/jbc.M114.572164 24878962 10.1074/jbc.M114.572164 PMC4139233

[22] Craven KB, Olivier NB, Zagotta WN. C-terminal movement during gating in cyclic nucleotide-modulated channels. J Biol Chem. 2008;283(21):14728–38. Epub 2008/03/28. doi: 10.1074/jbc.M710463200 ; PubMed Central PMCID: PMCPMC2386932. 18367452 10.1074/jbc.M710463200 PMC2386932

[23] Lolicato M, Bucchi A, Arrigoni C, Zucca S, Nardini M, Schroeder I, et al Cyclic dinucleotides bind the C-linker of HCN4 to control channel cAMP responsiveness. Nat Chem Biol. 2014;10(6):457–62. doi: 10.1038/nchembio.1521 24776929 10.1038/nchembio.1521

[24] Romling U, Galperin MY, Gomelsky M. Cyclic di-GMP: the first 25 years of a universal bacterial second messenger. Microbiol Mol Biol Rev. 2013;77(1):1–52. Epub 2013/03/09. doi: 10.1128/MMBR.00043-12 ; PubMed Central PMCID: PMCPMC3591986. 23471616 10.1128/MMBR.00043-12 PMC3591986

[25] Wu J, Sun L, Chen X, Du F, Shi H, Chen C, et al Cyclic GMP-AMP is an endogenous second messenger in innate immune signaling by cytosolic DNA. Science. 2013;339(6121):826–30. Epub 2012/12/22. doi: 10.1126/science.1229963 ; PubMed Central PMCID: PMCPMC3855410. 23258412 10.1126/science.1229963 PMC3855410

[26] Burdette DL, Monroe KM, Sotelo-Troha K, Iwig JS, Eckert B, Hyodo M, et al STING is a direct innate immune sensor of cyclic di-GMP. Nature. 2011;478(7370):515–8. Epub 2011/09/29. doi: 10.1038/nature10429 ; PubMed Central PMCID: PMCPMC3203314. 21947006 10.1038/nature10429 PMC3203314

[27] Brelidze TI, Carlson AE, Zagotta WN. Absence of direct cyclic nucleotide modulation of mEAG1 and hERG1 channels revealed with fluorescence and electrophysiological methods. J Biol Chem. 2009;284(41):27989–97. doi: 10.1074/jbc.M109.016337 19671703 10.1074/jbc.M109.016337 PMC2788851

[28] Brelidze TI, Carlson AE, Sankaran B, Zagotta WN. Structure of the carboxy-terminal region of a KCNH channel. Nature. 2012;481(7382):530–3. Epub 2012/01/11. doi: 10.1038/nature10735 ; PubMed Central PMCID: PMCPMC3267858. 22230959 10.1038/nature10735 PMC3267858

[29] Fivash M, Towler EM, Fisher RJ. BIAcore for macromolecular interaction. Curr Opin Biotechnol. 1998;9(1):97–101. 9503595 10.1016/s0958-1669(98)80091-8

[30] Malmqvist M. BIACORE: an affinity biosensor system for characterization of biomolecular interactions. Biochem Soc Trans. 1999;27(2):335–40. 10093759 10.1042/bst0270335

[31] Jorgensen TJ, Chen K, Chasovskikh S, Roy R, Dritschilo A, Uren A. Binding kinetics and activity of human poly(ADP-ribose) polymerase-1 on oligo-deoxyribonucleotide substrates. J Mol Recognit. 2009;22(6):446–52. doi: 10.1002/jmr.962 19585541 10.1002/jmr.962 PMC3493158

[32] Erkizan HV, Kong Y, Merchant M, Schlottmann S, Barber-Rotenberg JS, Yuan L, et al A small molecule blocking oncogenic protein EWS-FLI1 interaction with RNA helicase A inhibits growth of Ewing's sarcoma. Nat Med. 2009;15(7):750–6. doi: 10.1038/nm.1983 19584866 10.1038/nm.1983 PMC2777681

[33] Carlson AE, Brelidze TI, Zagotta WN. Flavonoid regulation of EAG1 channels. J Gen Physiol. 2013;141(3):347–58. doi: 10.1085/jgp.201210900 23440277 10.1085/jgp.201210900 PMC3581696

[34] Keller S, Vargas C, Zhao H, Piszczek G, Brautigam CA, Schuck P. High-precision isothermal titration calorimetry with automated peak-shape analysis. Anal Chem. 2012;84(11):5066–73. doi: 10.1021/ac3007522 22530732 10.1021/ac3007522 PMC3389189

[35] Zhao H, Piszczek G, Schuck P. SEDPHAT—a platform for global ITC analysis and global multi-method analysis of molecular interactions. Methods. 2015;76:137–48. doi: 10.1016/j.ymeth.2014.11.012 25477226 10.1016/j.ymeth.2014.11.012 PMC4380758

[36] Chow SS, Van PF, Accili EA. Energetics of cyclic AMP binding to HCN channel C terminus reveal negative cooperativity. J Biol Chem. 2012;287(1):600–6. doi: 10.1074/jbc.M111.269563 22084239 10.1074/jbc.M111.269563 PMC3249114

[37] Wang J, Chen S, Siegelbaum SA. Regulation of hyperpolarization-activated HCN channel gating and cAMP modulation due to interactions of COOH terminus and core transmembrane regions. J Gen Physiol. 2001;118(3):237–50. 11524455 10.1085/jgp.118.3.237 PMC2229504

[38] Wu S, Vysotskaya ZV, Xu X, Xie C, Liu Q, Zhou L. State-dependent cAMP binding to functioning HCN channels studied by patch-clamp fluorometry. Biophys J. 2011;100(5):1226–32. doi: 10.1016/j.bpj.2011.01.034 21354395 10.1016/j.bpj.2011.01.034 PMC3043209

[39] DeBerg HA, Brzovic PS, Flynn GE, Zagotta WN, Stoll S. Structure and Energetics of Allosteric Regulation of HCN2 Ion Channels by Cyclic Nucleotides. J Biol Chem. 2016;291(1):371–81. doi: 10.1074/jbc.M115.696450 26559974 10.1074/jbc.M115.696450 PMC4697172

[40] Moller S, Alfieri A, Bertinetti D, Aquila M, Schwede F, Lolicato M, et al Cyclic nucleotide mapping of hyperpolarization-activated cyclic nucleotide-gated (HCN) channels. ACS Chem Biol. 2014;9(5):1128–37. doi: 10.1021/cb400904s 24605759 10.1021/cb400904s

[41] Ng LC, Putrenko I, Baronas V, Van PF, Accili EA. Cyclic Purine and Pyrimidine Nucleotides Bind to the HCN2 Ion Channel and Variably Promote C-Terminal Domain Interactions and Opening. Structure. 2016;24(10):1629–42. doi: 10.1016/j.str.2016.06.024 27568927 10.1016/j.str.2016.06.024

[42] Milanesi R, Baruscotti M, Gnecchi-Ruscone T, DiFrancesco D. Familial sinus bradycardia associated with a mutation in the cardiac pacemaker channel. N Engl J Med. 2006;354(2):151–7. doi: 10.1056/NEJMoa052475 16407510 10.1056/NEJMoa052475

[43] Hammarstrom M, Hellgren N, van Den BS, Berglund H, Hard T. Rapid screening for improved solubility of small human proteins produced as fusion proteins in Escherichia coli. Protein Sci. 2002;11(2):313–21. doi: 10.1110/ps.22102 11790841 10.1110/ps.22102 PMC2373440

[44] Braun P, Hu Y, Shen B, Halleck A, Koundinya M, Harlow E, et al Proteome-scale purification of human proteins from bacteria. Proc Natl Acad Sci U S A. 2002;99(5):2654–9. doi: 10.1073/pnas.042684199 11880620 10.1073/pnas.042684199 PMC122403

[45] Lee CH, MacKinnon R. Structures of the Human HCN1 Hyperpolarization-Activated Channel. Cell. 2017;168(1–2):111–20. doi: 10.1016/j.cell.2016.12.023 28086084 10.1016/j.cell.2016.12.023 PMC5496774

[46] Kwan DC, Prole DL, Yellen G. Structural changes during HCN channel gating defined by high affinity metal bridges. J Gen Physiol. 2012;140(3):279–91. Epub 2012/08/30. doi: 10.1085/jgp.201210838 ; PubMed Central PMCID: PMCPMC3434101. 22930802 10.1085/jgp.201210838 PMC3434101

[47] Liao Z, Lockhead D, St Clair JR, Larson ED, Wilson CE, Proenza C. Cellular context and multiple channel domains determine cAMP sensitivity of HCN4 channels: ligand-independent relief of autoinhibition in HCN4. J Gen Physiol. 2012;140(5):557–66. doi: 10.1085/jgp.201210858 23109717 10.1085/jgp.201210858 PMC3483121

[48] Liao Z, Lockhead D, Larson ED, Proenza C. Phosphorylation and modulation of hyperpolarization-activated HCN4 channels by protein kinase A in the mouse sinoatrial node. J Gen Physiol. 2010;136(3):247–58. doi: 10.1085/jgp.201010488 20713547 10.1085/jgp.201010488 PMC2931151

[49] Vargas G, Lucero MT. Modulation by PKA of the hyperpolarization-activated current (Ih) in cultured rat olfactory receptor neurons. J Membr Biol. 2002;188(2):115–25. doi: 10.1007/s00232-001-0178-y 12172637 10.1007/s00232-001-0178-y

[50] Komagiri Y, Kitamura N. Comparison of effects of PKA catalytic subunit on I(h) and calcium channel currents in rat dorsal root ganglion cells. Biomed Res. 2007;28(4):177–89. 17878598 10.2220/biomedres.28.177

[51] Ingram SL, Williams JT. Modulation of the hyperpolarization-activated current (Ih) by cyclic nucleotides in guinea-pig primary afferent neurons. J Physiol. 1996;492 (Pt 1):97–106. 8730586 10.1113/jphysiol.1996.sp021292 PMC1158864

[52] Santoro B, Piskorowski RA, Pian P, Hu L, Liu H, Siegelbaum SA. TRIP8b splice variants form a family of auxiliary subunits that regulate gating and trafficking of HCN channels in the brain. Neuron. 2009;62(6):802–13. doi: 10.1016/j.neuron.2009.05.009 19555649 10.1016/j.neuron.2009.05.009 PMC2720631

[53] Zolles G, Wenzel D, Bildl W, Schulte U, Hofmann A, Muller CS, et al Association with the auxiliary subunit PEX5R/Trip8b controls responsiveness of HCN channels to cAMP and adrenergic stimulation. Neuron. 2009;62(6):814–25. doi: 10.1016/j.neuron.2009.05.008 19555650 10.1016/j.neuron.2009.05.008

[54] Bankston JR, Camp SS, DiMaio F, Lewis AS, Chetkovich DM, Zagotta WN. Structure and stoichiometry of an accessory subunit TRIP8b interaction with hyperpolarization-activated cyclic nucleotide-gated channels. Proc Natl Acad Sci U S A. 2012;109(20):7899–904. doi: 10.1073/pnas.1201997109 22550182 10.1073/pnas.1201997109 PMC3356637

[55] Nguyen HH, Park J, Kang S, Kim M. Surface plasmon resonance: a versatile technique for biosensor applications. Sensors (Basel). 2015;15(5):10481–510. Epub 2015/05/08. doi: 10.3390/s150510481 ; PubMed Central PMCID: PMCPMC4481982. 25951336 10.3390/s150510481 PMC4481982

[56] Campbell CT, Kim G. SPR microscopy and its applications to high-throughput analyses of biomolecular binding events and their kinetics. Biomaterials. 2007;28(15):2380–92. Epub 2007/03/06. doi: 10.1016/j.biomaterials.2007.01.047 . 17337300 10.1016/j.biomaterials.2007.01.047

[57] Arnold K, Bordoli L, Kopp J, Schwede T. The SWISS-MODEL workspace: a web-based environment for protein structure homology modelling. Bioinformatics. 2006;22(2):195–201. Epub 2005/11/23. doi: 10.1093/bioinformatics/bti770 . 16301204 10.1093/bioinformatics/bti770

