# Supplementary material for: Investigating cyclic nucleotide and cyclic dinucleotide binding to HCN channels by surface plasmon resonance
Source: PLoS One. 2017 Sep 26;12(9):e0185359. doi: 10.1371/journal.pone.0185359 (PMC5614581; doi:10.1371/journal.pone.0185359)
Supplement: S1 Fig — Identical residues are black on yellow background, similar residues are black on green background. The residues mutations of which altered cyclic dinucleotide response are indicated by arrows. Protein accession numbers are EDL31671 for mHCN2 and Q9Y3Q4 for hHCN4. (B) Structural alignment of the C-linker/CNBD of mHCN2 [15] (gray) and hHCN4 channels [14] (blue). cAMP bound inside the β-roll cavity is yellow and c-di-GMP placed in the proposed CLP site is cyan. (PDF) [file pone.0185359.s001.pdf]

A

C - linker

```
mHCN2 (443)  DSSRRQYQEKYKQVEQYMSFHKLPADFRQKIHDYYEHRYQ
hHCN4 (521)  DSSRRQYQEKYKQVEQYMSFHKLPPDTRQRIHDYYEHRYQ

mHCN2 (483)  GKMFDEDSILGELNGPLREEIVNFNCRKLVASMPLFANAD
hHCN4 (561)  GKMFDEESILGELSEPLREEIINFNCRKLVASMPLFANAD
```

CNBD

```
mHCN2 (523)  PNFVTAMLTKLKFEVFQPGDYIIREGTIGKKMYFIQHGVV
hHCN4 (601)  PNFVTSMLTKLRFEVFQPGDYIIREGTIGKKMYFIQHGVV

mHCN2 (563)  SVLTKGNKEMKLSDGSYFGEICLLTRGRRTASVRADTYCR
hHCN4 (641)  SVLTKGNKETKLADGSYFGEICLLTRGRRTASVRADTYCR

mHCN2 (603)  LYSLSVDNFNEVLEEYPMMRRAFETVAIDRLDRIGKKNSILLH
hHCN4 (681)  LYSLSVDNFNEVLEEYPMMRRAFETVALDRLDRIGKKNSILLH
```

B

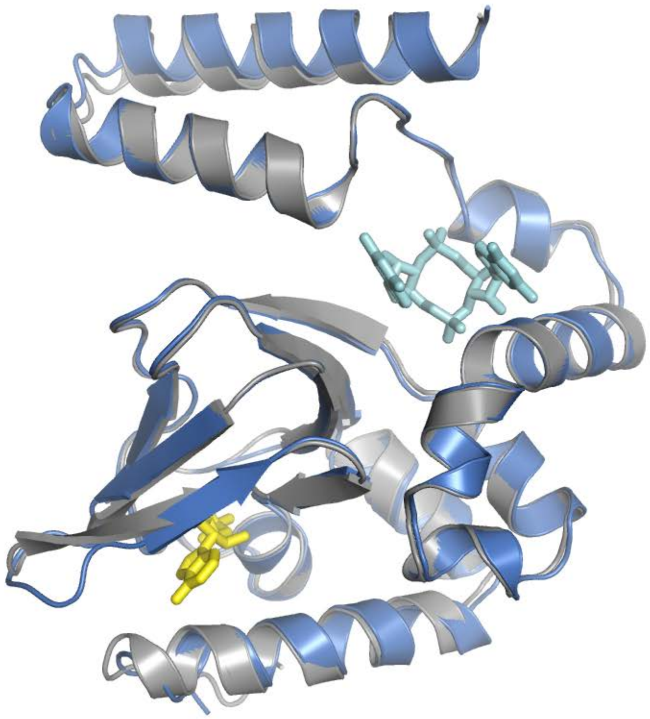

S1 Fig
