# Supplementary figures and images for: Investigating cyclic nucleotide and cyclic dinucleotide binding to HCN channels by surface plasmon resonance
Source: PLoS One. 2017 Sep 26;12(9):e0185359. doi: 10.1371/journal.pone.0185359 (PMC5614581; doi:10.1371/journal.pone.0185359)

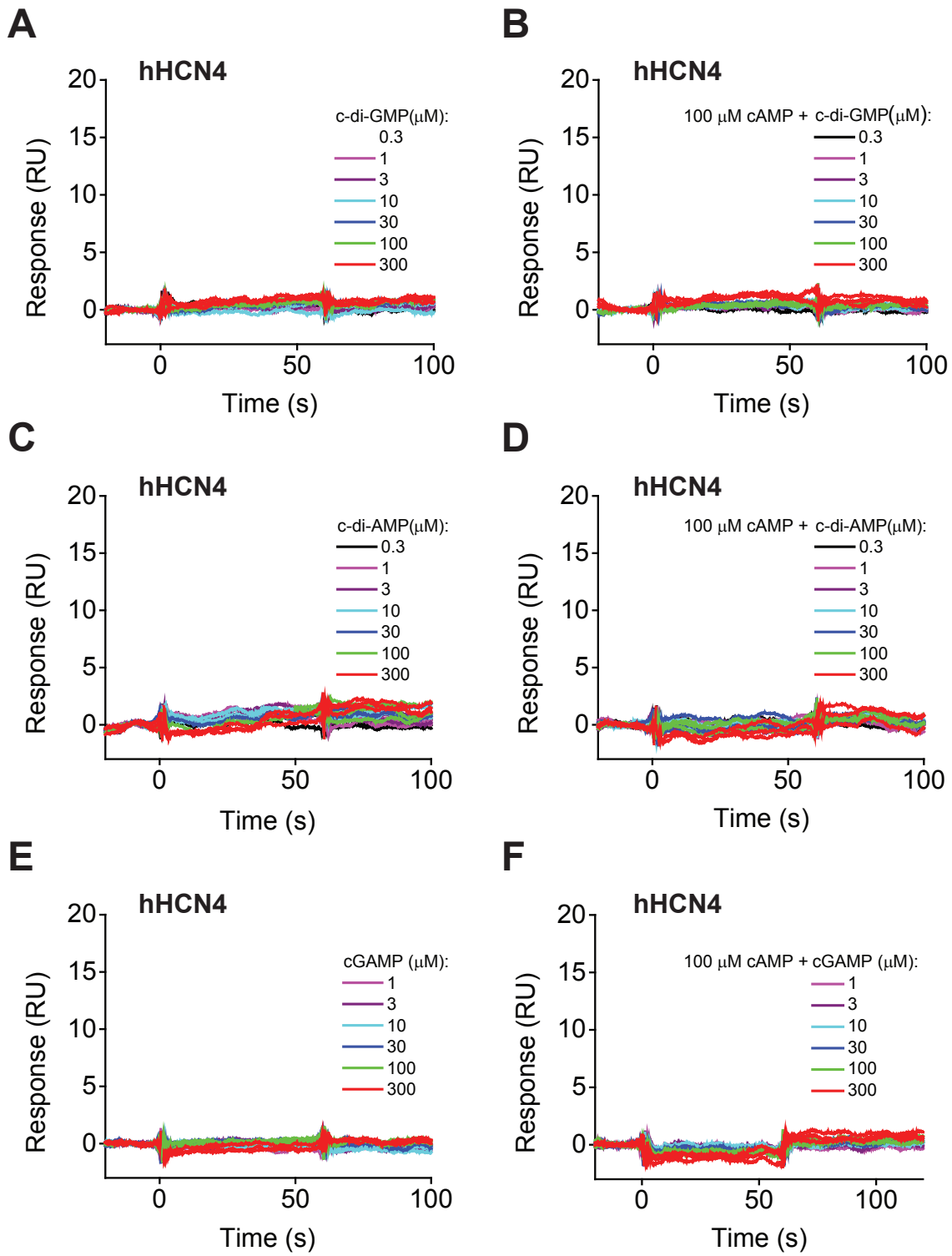

**S2 Fig**

Supplement: S2 Fig — Representative SPR sensorgrams recorded for the immobilized hHCN4 C-linker/CNBDs in the absence (A, C, E) and presence (B, D, F) of 100 μM cAMP with the indicated concentrations of c-di-GMP (A and B), c-di-AMP (C and D) and cGAMP (E and F). No increase in the binding response was detected upon injection of cyclic dinucleotides at the indicated concentration in the absence or presence of 100 μM cAMP. (PDF) [file pone.0185359.s002.pdf]
